# Identification, cloning and characterization of *sis7 *and *sis10 *sugar-insensitive mutants of *Arabidopsis*

**DOI:** 10.1186/1471-2229-8-104

**Published:** 2008-10-14

**Authors:** Yadong Huang, Chun Yao Li, Kelly D Biddle, Susan I Gibson

**Affiliations:** 1 Department of Plant Biology, University of Minnesota, 1500 Gortner Avenue, Saint Paul, MN 55108, USA; 2 Center for Technology in Teaching & Learning, MS-120, Rice University, 6100 Main Street, Houston, TX 77005, USA

## Abstract

**Background:**

The levels of soluble sugars, such as glucose and sucrose, help regulate many plant metabolic, physiological and developmental processes. Genetic screens are helping identify some of the loci involved in plant sugar response and reveal extensive cross-talk between sugar and phytohormone response pathways.

**Results:**

A forward genetic screen was performed to identify mutants with increased resistance to the inhibitory effects of high levels of exogenous sugars on early *Arabidopsis *seedling development. The positional cloning and characterization of two of these *sugar insensitive *(*sis*) mutants, both of which are also involved in abscisic acid (ABA) biosynthesis or response, are reported. Plants carrying mutations in *SIS7/NCED3/STO1 *or *SIS10/ABI3 *are resistant to the inhibitory effects of high levels of exogenous Glc and Suc. Quantitative RT-PCR analyses indicate transcriptional upregulation of ABA biosynthesis genes by high concentrations of Glc in wild-type germinating seeds. Gene expression profiling revealed that a significant number of genes that are expressed at lower levels in germinating *sis7-1/nced3-4/sto1-4 *seeds than in wild-type seeds are implicated in auxin biosynthesis or transport, suggesting cross-talk between ABA and auxin response pathways. The degree of sugar insensitivity of different *sis10/abi3 *mutant seedlings shows a strong positive correlation with their level of ABA insensitivity during seed germination.

**Conclusion:**

Mutations in the *SIS7/NCED3/STO1 *gene, which is primarily required for ABA biosynthesis under drought conditions, confer a sugar-insensitive phenotype, indicating that a constitutive role in ABA biosynthesis is not necessary to confer sugar insensitivity. Findings presented here clearly demonstrate that mutations in *ABI3 *can confer a sugar-insensitive phenotype and help explain previous, mixed reports on this topic by showing that ABA and sugar insensitivity exhibit a strong positive correlation in different *abi3 *mutants. Expression profiling revealed a potentially novel regulation of auxin metabolism and transport in an ABA deficient mutant, *sis7-1/nced3-4/sto1-4*.

## Background

Plant growth and development are regulated by signal transduction pathways that incorporate environmental stimuli and internal signals such as metabolic status. The absolute levels of soluble sugars, and/or the rate of flux of soluble sugars through certain metabolic pathways, are important indicators of metabolic status. Recent studies have established the role of sugars in regulating, at least partially, a variety of developmental processes from seed development and germination, early seedling development, vegetative growth and senescence to abiotic stress response [1-10].

Genetic screens based on the phenomenon that early seedling development of wild-type *Arabidopsis *can be arrested by high concentrations of exogenous sucrose (Suc) or glucose (Glc) have yielded sugar-response mutants. Characterization of these mutants has revealed that many also have defects in phytohormone metabolism or response [11-15]. In particular, several of these sugar-response mutants are allelic to abscisic acid (ABA) biosynthesis or response mutants such as *aba2*, *aba3 *or *abi4*. The sugar-resistant mutants *sis4 *[16], *gin1 *[15,17] and *isi4 *[18] are allelic to *aba2*, an ABA biosynthesis mutant [19]. The *gin5 *mutant [6,11] is allelic to another ABA deficient mutant, *aba3 *[19,20]. Similarly, mutations in *ABA1 *have been shown to confer Glc insensitivity [13]. The sugar-resistant mutants *sis5 *[16], *gin6 *[11], *isi3 *[18] and *sun6 *[13] are allelic to *abi4*, an ABA-insensitive mutant [21]. The *ABI4 *locus encodes an APETALA2 type transcription factor [22]. Mutations in *ABI5 *have also been shown to confer a weak, but still significant, sugar-insensitive phenotype [11,13,16]. Interestingly, plants carrying mutations in *ABI1 *or *ABI2 *have been found to exhibit a wild-type [11,16] or near wild-type [13], response to the inhibitory effects of high sugar concentrations on early seedling development. Previous research has given a mixed report on the role of ABI3 in sugar response. The *abi3-1 *mutant was found to exhibit an almost wild-type response to high levels of exogenous Glc [11,16] but was also reported to show a less pronounced but significant Glc insensitive phenotype when compared to wild-type plants [13]. In addition, it has been observed that specific *abi3 *alleles show decreased sensitivity to the inhibitory effects of a combination of ABA and Glc on seedling development [23]. Recently ABI3 has been reported to play an important role in inhibition of seed germination [24] and post-germinative growth [25] by Glc.

In screens of ethyl methane sulphonate (EMS)- or T-DNA mutagenized *Arabidopsis *populations for mutants with increased tolerance to the inhibitory effects of high levels of exogenous sugars on early seedling development, three *sugar insensitive *(*sis*) loci were identified. Map-based cloning experiments resulted in the identification of these loci. The *sis9-1 *mutation was found to lie in *ABA1*, a gene previously shown to act in ABA biosynthesis [26], and was not further characterized. The *sis7 *mutations lie in the same gene as the previously identified *nced3/sto1 *[27] mutants. The *sis10 *mutation lies in the same gene as the previously identified *abi3 *mutants [28]. *NCED3/STO1 *encodes a 9-*cis*-epoxycarotenoid dioxygenase [29]. Expression of *SIS7/NCED3/STO1 *has been suggested to be rate-limiting in the positive feedback regulation of ABA biosynthesis by ABA under stress conditions [17,26]. Expression profiling experiments identified 83 genes as being differentially regulated in the *sis7-1 *mutant when compared to wild-type seeds upon treatment with 100 mM Glc. *ABI3 *encodes a B3 domain transcription factor [30]. The sugar- and ABA-insensitivity of multiple *abi3 *mutant alleles show a strong positive correlation in our assays.

## Results

### Isolation of sugar-insensitive (*sis*) mutants *sis7 *and *sis10*

Most wild-type *Arabidopsis *seeds sown on minimal media [31] supplemented with high levels of Suc or Glc (e.g., 300 mM) germinate but the resulting seedlings fail to develop green expanded cotyledons and true leaves [11-13,15,16,32,33]. Instead, the seedlings develop small, white or purple cotyledons. In contrast, low concentrations (e.g., 30 mM) of exogenous Suc or Glc do not exert this inhibitory effect on normal shoot development. In addition, most wild-type seeds can develop into seedlings with expanded cotyledons and true leaves on media containing high concentrations (300 mM) of sorbitol, which acts as a non-metabolizable sugar analog in *Arabidopsis*. This observation indicates that the inhibitory effects of high sugar levels on early seedling development are not solely due to osmotic stress [16].

Genetic screens based on the inhibitory effects of high sugar levels on early seedling development led to the discovery of a number of sugar resistant mutants [11-13,15,16]. In one such screen performed previously by our lab, approximately 60,000 M2 seeds derived from an EMS-mutagenized *Arabidopsis thaliana *var. Col population were screened to identify seedlings that are resistant to 300 mM Suc. The mutants identified via this screen are also resistant to high concentrations of Glc and so were designated *sugar insensitive*, or *sis*, mutants [16]. Cloning and characterization of one of these mutants, the *sis7-2 *mutant, is described below. A similar mutant screen was conducted as part of the work reported here. This new mutant screen was conducted using a T-DNA mutagenized *Arabidopsis thaliana *var. Col population. The 35 SpBARN binary vector used to generate this population contains random *Arabidopsis *cDNAs driven by the Cauliflower Mosaic Virus 35S promoter [34]. Approximately 200,000 seeds from ~20,000 independent *Arabidopsis thaliana *var. Col transgenic lines were screened on media containing 340 mM Suc and ~1,700 seedlings that developed shoot systems with expanded cotyledons and true leaves were transferred to soil. This high rate of seedlings exhibiting an apparent sugar-resistant phenotype is due to the fact that some fraction (~1% in this experiment) of wild-type seedlings are able to escape the developmental arrest caused by high concentrations of exogenous sugars [16]. Therefore, it is necessary to screen seeds harvested from each putative mutant to identify the relatively small percentage that exhibit a heritable *sis *phenotype. Towards this end, the 1,700 seedlings were allowed to grow to maturity and seeds were harvested from each plant. Re-screening of seeds from each of the 1,700 putative mutants on media containing 340 mM Suc revealed that 18 of the putative mutants exhibit a reproducible *sis *phenotype. Characterization of these 18 mutants revealed that they appear to represent nine independent mutagenic events. The cloning and characterization of three of these new mutants, *sis7-1, sis7-3 *and *sis10-1*, as well as the *sis7-2 *mutant, is described below. The results of these studies indicate that the *sis7 *mutations lie in a gene previously shown to affect ABA metabolism, whereas the *sis10 *mutations lie in *ABI3*, which acts in ABA response [28].

### *sis7 *mutants exhibit a sugar-insensitive phenotype

**Figure 1:**
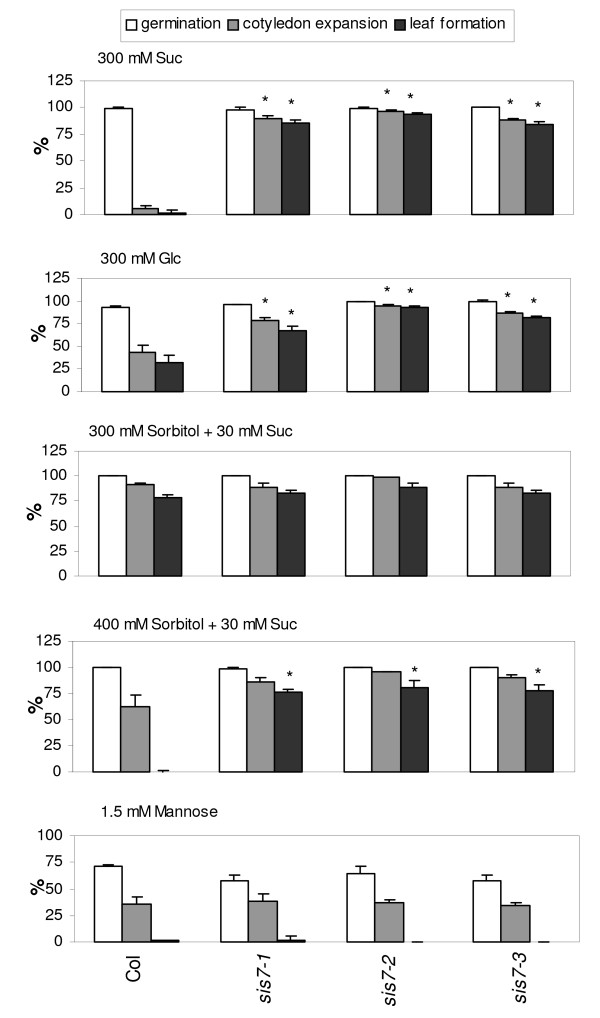
**Mutations in *SIS7 *confer a *sugar-insensitive *phenotype**. Col wild-type and *sis7 *seeds were stratified for 3 d and then sown on the indicated media and scored after 14 d at 22°C under continuous light. Data represent the means of three independent assays. The error bars indicate standard deviations. Asterisks indicate results where the mutant phenotype differed from the corresponding wild-type phenotype with a *p*-value of less than 0.05 according to a Student's t-test.

A significantly higher percentage of *sis7 *than of wild-type seeds develop into seedlings with relatively normal shoot systems on media containing 300 mM Suc or Glc (Figure 1). On media containing 300 mM Suc, more than 80% of all three *sis7 *mutants form expanded cotyledons and true leaves, whereas less than 6% of wild-type seedlings develop expanded cotyledons and true leaves. The mutants were also assayed for osmotic tolerance using media containing high concentrations of sorbitol. When assayed on equimolar (i.e. 300 mM) sorbitol supplemented with a low (30 mM) amount of Suc, differences are hard to detect between wild-type and mutant lines due to the fact that development of wild-type seedlings is not strongly affected at this concentration of sorbitol. In contrast, when assayed on media containing 400 mM sorbitol and 30 mM Suc, all three mutant alleles confer significantly increased osmotic resistance. Mannose (Man) is a Glc analogue that inhibits seed germination and early seedling development at millimolar concentrations via a mechanism that has been postulated to involve hexokinase [35]. Therefore, the mutants were assayed for Man sensitivity. None of the three *sis7 *mutant alleles tested cause a significant alteration in sensitivity to 1.5 mM Man compared to wild-type seedlings (Figure 1).

### *sis7-1 *exhibits resistance to the inhibition of seed germination by ABA and paclobutrazol

**Figure 2:**
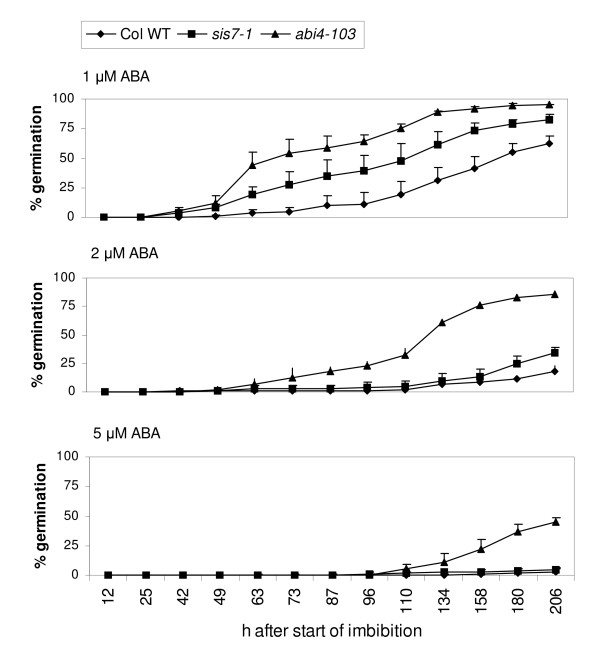
**The *sis7-1/sto1-4/nced3-4 *mutation confers a subtle ABA-insensitive phenotype**. Col wild-type and *sis7-1/sto1-4/nced3-4 *seeds were stratified for 3 d and then sown on media containing 1, 2 or 5 μM ABA and incubated at 25°C under continuous light conditions. Germination rates on minimal media were also checked and found to be consistently high, indicating a high rate of viability in the seeds used for these experiments (data not shown). Seed germination was scored at the indicated time points. Germination is defined as the emergence of the radicle from the seed coat. Data represent the means of three independent assays. The error bars represent standard deviations. The *abi4-103/sis5-3 *[16] seeds were included as a positive control.

**Figure 3:**
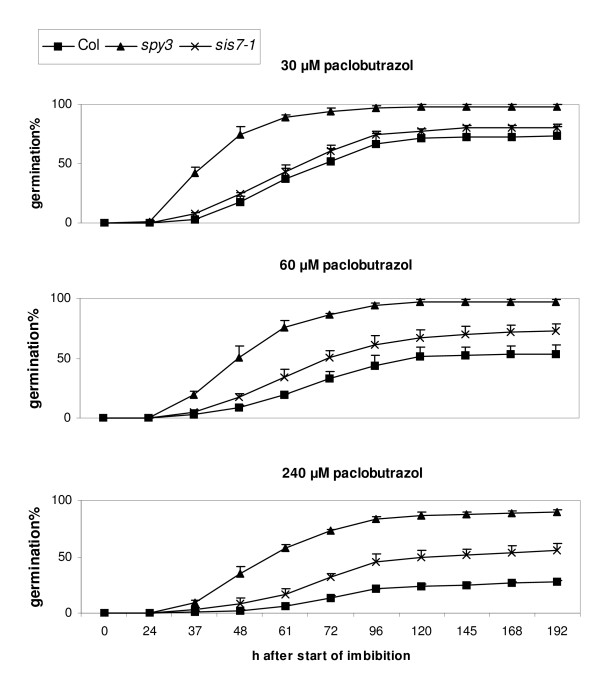
**The *sis7-1/sto1-4/nced3-4 *mutation confers a paclobutrazol-resistant phenotype**. Col wild-type and *sis7-1/sto1-4/nced3-4 *seeds were stratified for 3 d and then sown on media containing 30, 60 or 240 μM paclobutrazol and incubated at 25°C under continuous light conditions. Germination rates on minimal media were also checked and found to be consistently high, indicating a high rate of viability in the seeds used for these experiments (data not shown). Seed germination was scored at the indicated time points. Germination is defined as the emergence of the radicle from the seed coat. Data represent the means of three independent assays. The error bars represent standard deviations. The *spy-3 *[36] seeds were included as a positive control.

Previous studies have shown that *sis4/aba2 *mutants are resistant to the inhibitory effects of paclobutrazol, an inhibitor of gibberellin biosynthesis, on seed germination. Similarly, *sis5/abi4 *mutants are resistant to the inhibitory effects of both paclobutrazol and ABA on seed germination [16]. To determine whether mutations in *SIS7 *exert similar effects, the germination rates of *sis7-1 *seeds on media containing ABA or paclobutrazol were determined. Seeds carrying the *sis7-1 *mutation exhibit significantly faster germination rates than wild-type seeds on media containing 1 μM ABA and slightly faster germination rates on 2 μM ABA, but have wild-type germination rates on higher concentrations (e.g. 5 μM) of ABA (Figure 2). Germination rates of *sis7-1 *and wild-type seeds were also assayed on media containing several different concentrations of paclobutrazol. The *spy3 *seeds were included as a positive control as they have previously been shown to be resistant to paclobutrazol [36]. Seeds carrying the *sis7-1 *mutation display enhanced germination rates compared to wild-type seeds on media containing high levels (e.g. 60 or 240 μM) of paclobutrazol (Figure 3).

### Cloning of *SIS7 *and pharmacological complementation of the *sis7 *phenotype

**Figure 4:**
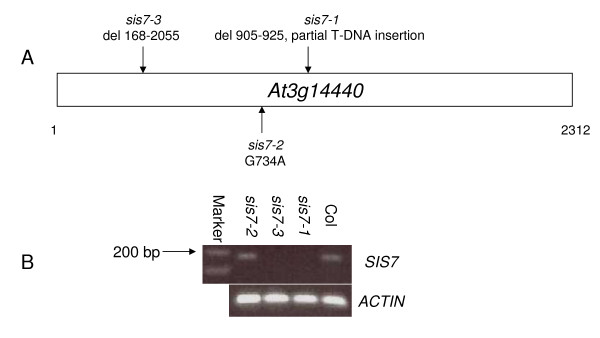
**Characterization of *sis7 *molecular lesions**. (A) Mutations in *SIS7/NCED3/STO1*. The molecular lesions associated with the three *sis7/nced3/sto1 *mutants identified as part of this work are shown. Nucleotides are numbered with respect to the start codon. (B) *SIS7 *transcript levels in wild-type and *sis7 *plants. RT-PCR was used to amplify *SIS7*/*NCED3*/*STO1 *transcripts from 4-week old wild-type (Col) and *sis7 *mutant plants. 2 μL cDNA was used as a template for the first PCR reaction (35 cycles). *ACTIN *transcripts were amplified as a positive control.

A map-based cloning approach was used to identify the *SIS7 *gene. The *sis7-1 *mutant, which is in the Col background, was crossed to wild-type Hi-O plants. F2 progeny of this cross were screened on media containing 340 mM Suc. Those seedlings that formed relatively normal shoot systems (i.e. displayed a *sis *mutant phenotype) were selected and used to form a mapping population of 940 plants. SSLP and CAPS markers were used to localize *sis7 *to a 78 kb region between BAC clones MLN21 and MIE1 on chromosome 3. DNA spanning the entire 78 kb region was isolated from *sis7-1 *by PCR and sequenced. Analysis of this sequencing data reveals that *sis7-1 *carries a 21 bp deletion and a partial T-DNA insertion in the exon region of At3g14440. No other mutations were found in the region shown by mapping to contain the *sis7-1 *mutation. Another *sis *mutation was independently mapped to chromosome 3 between BACs MLN21 and MSJ11, the region which contains the At3g14440 gene. Sequencing of the putative *SIS7 *gene from this mutant, now named *sis7-2*, identified a point mutation in the exon region that changes amino acid codon 245 from Gly to Asp. Sequencing of the putative *SIS7 *gene from a third *sis *mutant revealed that this mutant, now named *sis7-3*, contains a large deletion in the *SIS7 *gene (Figure 4A). The finding that three independent *sis *mutants all carry mutations in At3g14440 provides convincing evidence that the mutations in this gene are responsible for the *sis *phenotype observed in these lines. At3g14440 has previously been shown to encode 9-*cis*-epoxycarotenoid dioxygenase (NCED3), a key enzyme in the biosynthesis of ABA [29,37]. Plants carrying a T-DNA insertion in *NCED3 *are drought sensitive [29]. An additional mutant of *NCED3*, named *sto1*, was identified in a screen for salt-tolerant mutants. These *sto1 *mutants exhibit resistance to the inhibitory effects of hyperosmotic stress on seed germination, but not on post-germinative growth [27]. A third mutant carrying a T-DNA insertion in *NCED3 *has also been described [38]. The three mutant alleles identified in this study were accordingly re-designated *sis7-1/nced3-4/sto1-4, sis7-2/nced3-5/sto1-5 *and *sis7-3/nced3-6/sto1-6*. To determine whether *SIS7 *mRNA levels are altered in the *sis7 *mutants, *SIS7 *transcript abundance was assayed by RT-PCR in wild-type plants and in all three *sis7 *mutants. *SIS7 *transcript was not detected in *sis7-1 *or *sis7-3*, whereas in *sis7-2 SIS7 *transcript abundance is approximately the same as in wild-type plants (Figure 4B).

**Figure 5:**
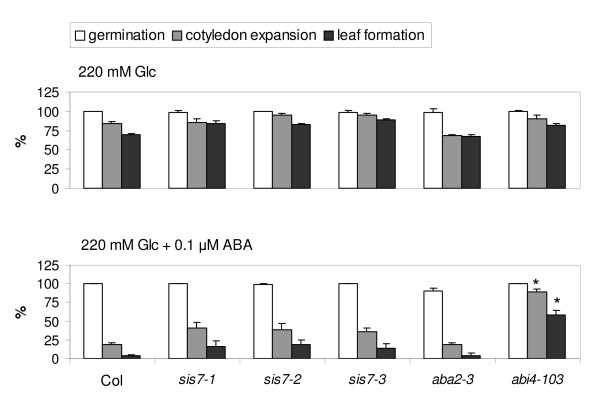
**Exogenous ABA causes *sis7 *mutants to exhibit approximately wild-type sugar sensitivity**. Wild-type (Col) and mutant seeds were cold treated for 3 d and then sown on minimal media supplemented with 220 mM Glc, with or without 0.1 μM ABA. Additional mutants deficient in ABA biosynthesis (*aba2-3*) or ABA response (*abi4-103*) were included as controls. Percent seed germination, cotyledon expansion and true leaf formation were scored after an additional 14 d incubation under continuous light at 22°C. Data represent the means of three independent assays. The error bars represent standard deviations. Asterisks indicate results where the phenotype of one of the mutants differed from that of the wild type with a *p*-value of less than 0.05 according to a Student's t-test.

Consistent with other *aba *mutants, *sis7-1/nced3-4/sto1-4 *exhibits a wilty phenotype when subject to water deprivation (data not shown). To determine if the sugar insensitivity of the *sis7/nced3/sto1 *mutants is affected by ABA deficiency, pharmacological complementation of *sis7/nced3/sto1 *mutant seedlings was conducted as described [11,17]. Early seedling development of Col wild type, three *sis7 *mutant alleles, the ABA deficient mutant *aba2-3 *and an ABA insensitive mutant *abi4-103 *were not affected by 220 mM Glc media. Addition of a noninhibitory level of 0.1 μM ABA to the media containing 220 mM (4%) Glc restored Glc sensitivity of *sis7-1/nced3-4/sto1-4, sis7-2/nced3-5/sto1-5 *and *sis7-3/nced3-6/sto1-6*, as well as of the ABA deficient mutant *aba2-3*, but not that of the ABA insensitive mutant *abi4-103 *(Figure 5). These results indicate that the *sis *phenotype of all three *sis7 *mutant alleles is caused by ABA deficiency.

### Glc regulation of ABA biosynthesis gene expression in germinating seeds

**Figure 6:**
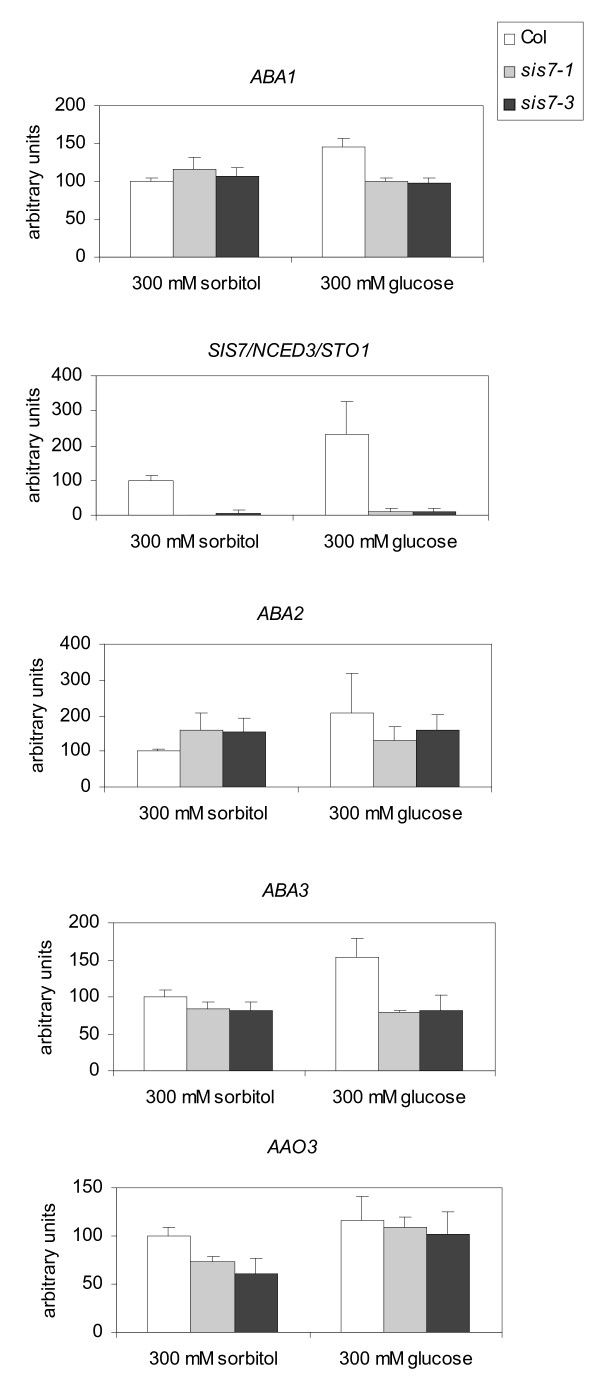
**Quantitative RT-PCR analysis of ABA biosynthesis genes in *sis7 *and wild-type germinating seeds**. Relative expression levels were measured by qRT-PCR in Col, *sis7-1 *and *sis7-3 *germinating seeds treated with 300 mM Glc or 300 mM sorbitol. Data were obtained from two biologically independent experiments and relative mRNA levels were determined by using *UBQ6 *as a reference. The relative mRNA level of each gene in sorbitol-treated Col was set to 100. Bars indicate standard deviations.

Previously it has been shown that 7% Glc causes ABA accumulation (or decreased metabolism) in wild-type seedlings and that an increase in ABA levels is required for Glc-dependent developmental arrest [11]. The expression levels of the ABA biosynthesis genes, *ABA1*, *ABA2*, *AAO3 *and *ABA3 *were found to be increased by 2 and 6% Glc. In contrast, *NCED3 *expression was not activated by Glc [17]. However, as these studies were conducted using young seedlings, the mechanism of high Glc-induced ABA accumulation in germinating seeds remains unclear. Furthermore, it has been shown that high concentrations of exogenous sugars inhibit early seedling development only during a narrow temporal window (less than 40 hours after the start of imbibition) [12]. We therefore performed quantitative real-time PCR (qRT-PCR) analysis of ABA biosynthesis gene expression in both *sis7 *and wild-type germinating seeds. In brief, two biologically independent batches of Col wild-type, *sis7-1 *and *sis7-3 *seeds were sown on minimal media for 20 h and transferred to media supplemented with 300 mM Glc or 300 mM sorbitol for 13 h under continuous light before harvest. The total time of treatment was within the temporal window during which exogenous sugars can arrest seedling development [12]. Total RNA was extracted for qRT-PCR analysis. As shown in Figure 6, *ABA1*, *SIS7/NCED3*, *ABA2 *and *ABA3 *exhibit higher steady-state mRNA levels in wild-type seeds germinating in the presence of 300 mM Glc than in the presence of 300 mM sorbitol. In contrast, *AAO3 *steady-state mRNA levels are not significantly different in wild-type seeds germinating on Glc than on sorbitol. Glc induction of *SIS7/NCED3*, as well as *ABA1, ABA2 *and *ABA3*, is abolished in the *sis7-1 *and *sis7-3 *null mutants, suggesting a certain level of endogenous ABA may be required for Glc induction of ABA biosynthesis gene expression in germinating seeds.

### Transcriptional profiling identifies mis-regulated genes in the *sis7/nced3/sto1 *mutant

To identify genes that are differentially expressed in *sis7-1/nced3-4/sto1-4 *versus wild type, *Arabidopsis *Affymetrix ATH1 GeneChips were used for transcriptional profiling of germinating *sis7-1/nced3-4/sto1-4 *and wild-type seeds. Col wild-type and *sis7-1/nced3-4/sto1-4 *seeds were sown on minimal media for 20 h and transferred to 100 mM Glc or equimolar sorbitol (as an osmotic control) media for 13 h under continuous light to allow induction or repression of Glc-responsive genes. At the end of this incubation period 0–4% of the seeds had germinated. The reason that seeds were collected after a total of 33 h is based on the observation that exogenous sugars inhibit seedling development during a narrow temporal window (less than 40 hours after start of imbibition) [12]. Data analysis using the Expressionist software package is as detailed in "Materials and methods". To identify genes that are expressed at significantly different levels in *sis7-1/nced3-4/sto1-4 *versus wild-type germinating seeds grown in the presence of 100 mM Glc, the results of three independent GeneChip experiments using mutant seeds were compared with the results of six independent biological replicates of wild-type samples. Significant differences in expression values were defined as those where the average expression levels between mutant and wild-type seeds differ by at least two fold and have a Student's t-test *p *value of less than 0.05. Using these cutoffs, the levels of 83 transcripts were found to be altered in *sis7-1/nced3-4/sto1-4 *versus wild-type seeds incubated in the presence of 100 mM Glc (Table 1). Of these genes, 20 had higher mRNA levels in the mutant than in the wild type, whereas 63 genes had lower mRNA levels in the mutant.

**Table 1:** Genes with altered expression in *sis7-1 *versus wild-type (WT) seeds incubated on 100 mM Glc media.

Description	AGI	*sis7-1*	WT	sis7-1/WT	*P*-Value
genes with higher transcript levels in *sis7-1*					
expressed protein	AT5G58375	116965	3030	38.60	9.47E-09
2S seed storage protein 4	AT4G27170	15028	874.6	17.18	0.002
cytosolic small heat shock protein	AT5G12030	6968	977.5	7.13	0.020
protease inhibitor/seed storage family protein	AT5G54740	9475	1824	5.19	0.033
heat shock protein 70, putative	AT3G12580	36044	9615	3.75	0.016
expressed protein	AT2G32210	5544	1555	3.56	0.004
gibberellin-responsive protein 3	AT4G09600	2995	1066	2.81	0.008
alternative oxidase 1a, mitochondrial (AOX1A)	AT3G22370	4803	1982	2.42	0.024
low temperature and salt responsive protein	AT4G30650	4272	1769	2.42	0.029
putative GTP-binding protein	AT1G09180	1816	758.1	2.40	0.026
AP2 domain transcription factor	AT2G40350	1848	787.9	2.35	0.031
steroid 5alpha-reductase-like protein	AT5G16010	10002	4287	2.33	0.001
expressed protein	AT5G24570	3708	1594	2.33	0.028
arabinogalactan-protein	AT5G64310	33863	14633	2.31	0.011
CDC48 – like protein	AT3G53230	21244	9218	2.30	0.007
pathogenesis-related thaumatin family protein	AT4G36010	10479	4607	2.27	0.013
mitochondrial heat shock 22 kd protein-like	AT5G51440	3154	1492	2.11	0.041
expressed protein	AT5G64230	2056	982.4	2.09	0.026
glycine-rich protein/oleosin	AT3G18570	3873	1872	2.07	0.006
salt-tolerance zinc finger protein	AT1G27730	4463	2200	2.03	0.017
					
genes with lower transcript levels in *sis7-1*					
cytochrome P450 79B2, putative (CYP79B2)	AT4G39950	1389	7597	0.18	0.001
RuBisCO activase	AT2G39730	1489	4706	0.32	0.007
auxin-responsive GH3 family protein	AT4G27260	2324	6679	0.35	0.047
cytochrome P450 83B1 (CYP83B1)	AT4G31500	15681	44352	0.35	0.003
S1 RNA-binding domain-containing protein	AT1G12800	1259	3475	0.36	0.007
pectin methylesterase	AT1G11580	4380	11956	0.37	0.009
DegP2 protease	AT2G47940	1759	4776	0.37	0.007
AMP-binding protein	AT3G23790	878	2291	0.38	0.040
putative activator subunit of SNF1-related protein kinase, SNF4	AT1G09020	1759	4584	0.38	0.035
WPP-domain interacting protein 3	AT3G13360	627	1614	0.39	0.014
MATE efflux family protein	AT1G71870	1830	4621	0.40	0.016
glutathione S-transferase	AT2G30860	36776	92522	0.40	0.002
hydrolase family protein	AT2G25870	981	2438	0.40	0.000
endo-beta-1,4-glucanase	AT1G64390	2208	5381	0.41	0.011
heat shock protein 100, putative	AT5G15450	1817	4398	0.41	0.026
eIF-2 family protein	AT1G76810	748	1809	0.41	0.035
auxin transport protein, putative (PIN3)	AT1G70940	1147	2735	0.42	0.009
male sterility MS5 family protein	AT5G48850	1109	2638	0.42	0.032
phosphatidylinositolglycan class family protein	AT3G01380	1150	2691	0.43	0.006
epsilon-adaptin, putative	AT1G31730	1767	4134	0.43	0.016
PSII low MW protein	ATCG00220	11808	27598	0.43	0.026
protein kinase family protein	AT1G75640	393	897.5	0.44	0.021
guanylate-binding family protein	AT5G46070	607	1382	0.44	0.018
proteaseI (pfpI)-like protein (YLS5)	AT2G38860	4039	9166	0.44	0.008
sulfotransferase family protein	AT1G74100	11829	26477	0.45	0.002
chlorophyll A-B binding protein 4, chloroplast	AT3G47470	532	1175	0.45	0.042
senescence-associated protein-related	AT5G20700	633	1395	0.45	0.028
early dehydration-induced gene ERD13	AT2G30870	3684	8114	0.45	0.046
squamosa promoter-binding protein-like 1	AT2G47070	976	2147	0.45	0.011
heavy-metal-associated domain protein	AT5G19090	1141	2502	0.46	0.010
expressed protein	AT3G50370	1209	2652	0.46	0.004
methyltransferase MT-A70 family protein	AT4G09980	850	1840	0.46	0.005
SET domain-containing protein	AT2G19640	2024	4381	0.46	0.008
UV-damaged DNA-binding protein, putative	AT4G05420	2387	5151	0.46	0.000
expressed protein	AT4G27450	7573	16323	0.46	0.010
zinc finger protein-related	AT3G18290	1140	2444	0.47	0.005
uroporphyrinogen decarboxylase, putative	AT2G40490	1408	2997	0.47	0.017
molybdenum cofactor sulfurase family protein	AT5G44720	1619	3427	0.47	0.020
expressed protein	AT1G36990	1152	2437	0.47	0.008
kinesin motor family protein	AT5G66310	255	538.8	0.47	0.019
pentatricopeptide repeat-containing protein	AT1G71060	419	883	0.47	0.035
protein kinase-related	AT3G03930	441	927.2	0.48	0.008
exportin1 (XPO1)	AT5G17020	2877	6035	0.48	0.010
auxin efflux carrier protein, putative (PIN1)	AT1G73590	2463	5154	0.48	0.021
pentatricopeptide repeat-containing protein	AT5G46580	3046	6365	0.48	0.007
pentatricopeptide repeat-containing protein	AT5G28370	3099	6453	0.48	0.019
expressed protein	AT5G63135	902	1875	0.48	0.011
male sterility MS5 family protein	AT1G04770	2548	5292	0.48	0.004
MAPK, putative (MPK8)	AT1G18150	749	1541	0.49	0.046
expressed protein	AT5G64460	769	1580	0.49	0.043
importin beta-2 subunit family protein	AT2G31660	2429	4987	0.49	0.007
defense-related protein, putative	AT4G30530	3242	6650	0.49	0.003
calcium-transporting ATPase 4	AT2G41560	6059	12419	0.49	0.001
ubiquitin-transferase family protein	AT4G38600	5327	10886	0.49	0.011
sulfite reductase/ferredoxin (SIR)	AT5G04590	4299	8781	0.49	0.020
VARIEGATED 3	AT5G17790	646	1317	0.49	0.005
heat shock protein, putative	AT1G11660	1120	2280	0.49	0.030
pentatricopeptide repeat-containing protein	AT2G17033	716	1452	0.49	0.013
nuclease family protein	AT5G61780	8110	16406	0.49	0.027
pentatricopeptide repeat-containing protein	AT2G31400	3807	7676	0.50	0.040
patched family protein	AT4G38350	2104	4230	0.50	0.015
Involved in the biosynthesis of brassinosteroids	AT2G07050	3755	7538	0.50	0.022
ATP phosphoribosyl transferase	AT1G09795	1402	2805	0.50	0.000

**Figure 7:**
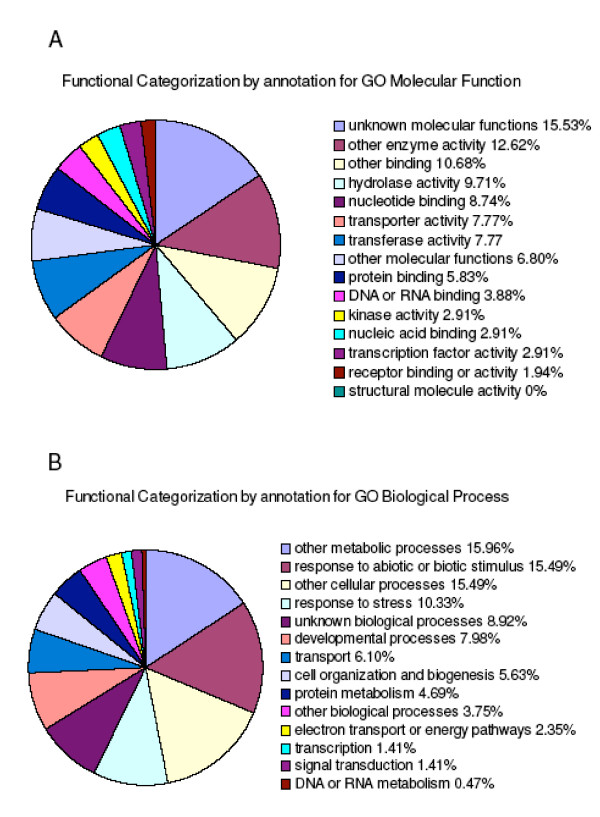
**Functional categorization of genes mis-regulated in *sis7-1 *on 100 mM Glc**. Affymetrix ATH1 GeneChips were used for transcriptional profiling of germinating *sis7-1/nced3-4/sto1-4 *and wild-type seeds. Col wild-type and *sis7-1/nced3-4/sto1-4 *seeds were sown on minimal media for 20 h and transferred to 100 mM Glc media for 13 h under continuous light. The Expressionist software package was used to identify genes with transcript levels that differ by at least two fold and with a *p *value of less than 0.05 when comparing the results of mutant versus wild-type samples. Using these cutoffs, the levels of 83 transcripts were found to be altered in *sis7-1/nced3-4/sto1-4 *versus wild-type seeds (Table 1). Functional categorization of these mis-regulated genes was performed using the GO (Gene Ontology) Annotations tool from TAIR [39]. Panel A shows the distribution of annotations for molecular function and panel B shows the distribution for annotations for biological process. Percentages were calculated as: (number of annotations to terms in this GOslim category/number of total annotations to terms in this ontology) × 100.

Functional categorization of these mis-regulated genes was performed using the Gene Ontology (GO) Annotations tool from TAIR [39] and the results are presented in Figure 7. Notably, a significant percentage of the mis-regulated genes are involved in response to abiotic or biotic stimulus and to stress (a combined 25.82%). Over-representation analysis (ORA) was also performed. ORA is a useful tool for analyzing gene expression data obtained from microarray experiments as it can determine whether a set of genes is statistically over-represented among the total genes expressed on the microarray. An online version of the GeneMerge program [40] was used for ORA to identify whether particular biological processes or molecular functions, as defined by GO annotations, are statistically enriched in the set of differentially expressed genes in the *sis7-1 *mutant compared to the wild type [41]. Genes involved in response to stimulus (GO:0050896) are significantly enriched (16/83; *p *= 0.0095 after Bonferroni correction for multiple tests) and genes involved in response to abiotic stimulus (GO:0009628, a child term of GO:0050896) are also significantly over-represented (10/83; *p *= 0.0167). Both of these sets of genes include *PIN1*, *PIN3 *and *CYP83B1*. Manual inspection of the data identified two other genes involved in auxin metabolism: *CYP79B2 *and At4g27260. Thus, interestingly, genes involved in auxin biosynthesis and transport represent 6% of the genes expressed at altered levels in *sis7-1 *versus wild-type seeds grown on 100 mM Glc, and so are significantly over-represented amongst these genes, as auxin-related genes comprise far less than 6% of the *Arabidopsis *genome. *CYP79B2 *is involved in Trp metabolism and converts Trp to indole-3-acetaldoxime (IAOx), a precursor to indole acetic acid (IAA) [42,43]. Expression of *CYP79B2 *is reduced more than five-fold in mutant seeds relative to wild-type seeds grown in the presence of 100 mM Glc. At4g27260 encodes an IAA-amido synthase that conjugates Asp and other amino acids to auxin *in vitro*. An insertional mutation in this gene causes increased sensitivity to IAA in seedling roots [44]. *CYP83B1 *is required for red light signal transduction and auxin homeostasis [45]. *PIN1 *and *PIN3 *are auxin efflux transporters and regulate root development [46]. Interplay between auxin and ABA signaling to regulate lateral root development has been suggested by previous studies [47-49]. The decreased mRNA levels of these auxin-related genes in Glc-treated *sis7-1/nced3-4/sto1-4 *seeds provide further evidence of cross-talk between these pathways.

**Figure 8:**
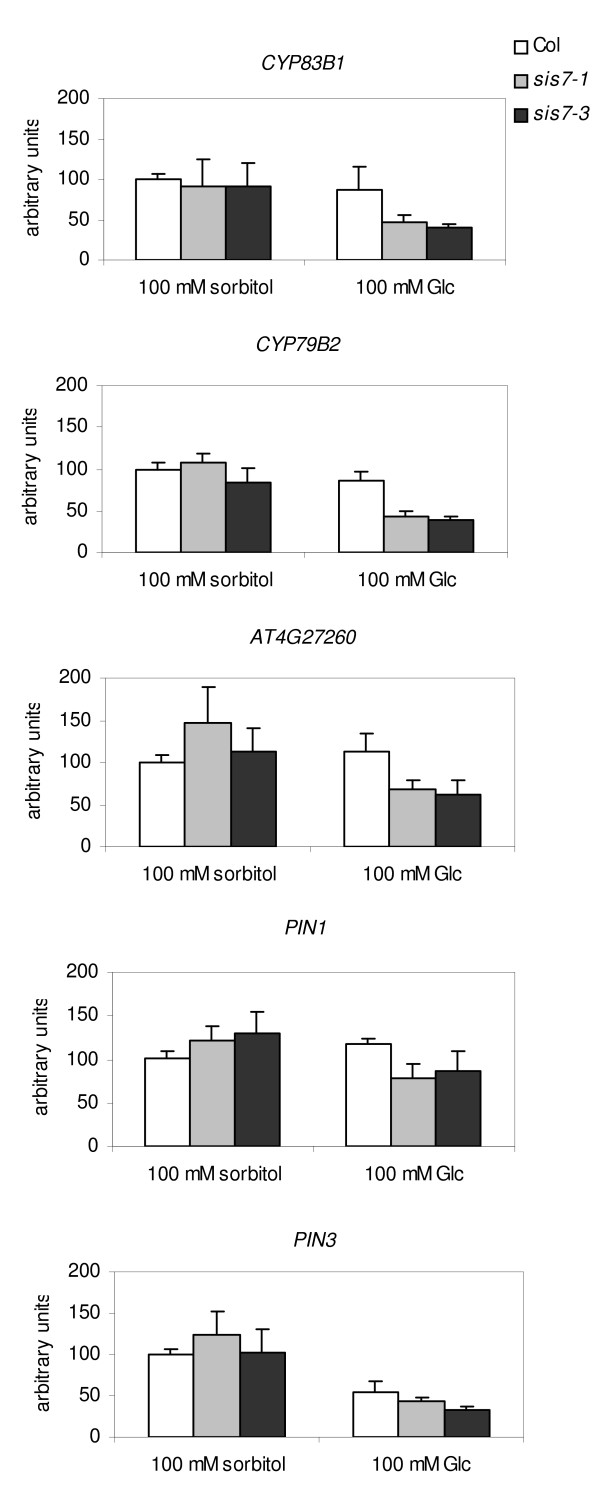
**Quantitative RT-PCR analysis of auxin-related genes**. Genes involved in auxin metabolism or transport were chosen for analysis. Relative expression levels of these genes were measured by qRT-PCR in wild-type Col, *sis7-1 *and *sis7-3 *germinating seeds treated with 100 mM Glc or 100 mM sorbitol. Data were obtained from two biologically independent experiments and relative mRNA levels were determined by using *UBQ6 *as a reference. The relative mRNA level of each gene in sorbitol-treated wild-type Col was set to 100. Bars indicate standard deviations.

To determine which of the genes listed in Table 1 are glucose regulated, the data from GeneChip experiments performed using seeds grown in the presence of 100 mM Glc were compared with data from GeneChip experiments performed using seeds grown in the presence of equimolar sorbitol. Based on these comparisons, the fold change in expression level (Glc/sorbitol) was determined for each of the genes listed in Table 1 for both *sis7-1 *and wild-type seeds (Table 2). Based on their glucose-responsiveness in *sis7-1 *and wild type, these genes can be classified into three categories. The first category includes genes that show similar glucose induction or repression in both *sis7-1 *and wild type. For example, the photosynthesis-related genes encoding CAB4 and RuBisCo activase are repressed by 100 mM Glc in wild-type as well as in *sis7-1 *seeds (Table 2). Similarly, the expression of *PIN3 *is Glc-repressed in both *sis7-1 *and wild type (Table 2 and Figure 8). The second category represents the majority of the genes on the list, which are not significantly regulated by Glc in either wild-type or *sis7-1 *seeds. The third category contains a few genes that are Glc-regulated in *sis7-1 *but that exhibit little to no Glc regulation in wild type. For example, *At4g30650 *transcript levels are increased 3.6 fold by 100 mM Glc in *sis7-1 *seeds but only 1.3 fold in wild-type seeds. Similarly, the expression of *CYP79B2 *is repressed 2-fold by Glc in *sis7-1 *seeds but only 1.4 fold in wild-type seeds. At5g46070 transcript levels are decreased approximately 2-fold by 100 mM Glc in *sis7-1 *seeds but are unchanged by Glc in wild-type seeds (Table 2 and Figure 8). The identification of this third category of genes, combined with the fact that *sis7-1 *seeds are ABA-deficient [27,29], suggests that the Glc-responsiveness of the genes in the third category is affected by endogenous ABA levels.

**Table 2:** Glc-responsiveness of genes identified in Table I in *sis7-1 *and wild-type (WT) seeds. ND = not determined.

AGI	Description	*sis7-1 *glc/sorbitol	WT glc/sorbitol
AT1G04770	male sterility MS5 family protein	0.54	0.64
AT1G09020	putative activator subunit of SNF1-related protein kinase, SNF4	0.35	0.69
AT1G09180	putative GTP-binding protein	0.94	1.54
AT1G09795	ATP phosphoribosyl transferase	0.62	1.11
AT1G11580	pectin methylesterase	0.21	0.38
AT1G11660	heat shock protein, putative	0.82	1.20
AT1G12800	S1 RNA-binding domain-containing protein	0.55	0.84
AT1G18150	MAPK, putative (MPK8)	0.52	0.93
AT1G27730	salt-tolerance zinc finger protein	3.28	2.04
AT1G31730	epsilon-adaptin, putative	0.51	0.85
AT1G36990	expressed protein	0.56	0.81
AT1G64390	endo-beta-1,4-glucanase	0.39	0.65
AT1G70940	auxin transport protein, putative (PIN3)	0.53	0.66
AT1G71060	pentatricopeptide repeat-containing protein	0.64	0.92
AT1G71870	MATE efflux family protein	0.36	0.69
AT1G73590	auxin efflux carrier protein, putative (PIN1)	0.77	1.09
AT1G74100	sulfotransferase family protein	0.73	0.89
AT1G75640	protein kinase family protein	0.57	1.10
AT1G76810	eIF-2 family protein	0.64	1.05
AT2G07050	Involved in the biosynthesis of brassinosteroids	0.66	0.99
AT2G17033	pentatricopeptide repeat-containing protein	0.61	0.71
AT2G19640	SET domain-containing protein	0.51	0.93
AT2G25870	hydrolase family protein	0.63	1.14
AT2G30860	glutathione S-transferase	0.70	0.91
AT2G30870	early dehydration-induced gene ERD13	0.91	1.08
AT2G31400	pentatricopeptide repeat-containing protein	0.53	0.73
AT2G31660	importin beta-2 subunit family protein	0.76	1.11
AT2G32210	expressed protein	3.32	1.93
AT2G38860	proteaseI (pfpI)-like protein (YLS5)	1.02	1.17
AT2G39730	RuBisCO activase	0.42	0.52
AT2G40350	AP2 domain transcription factor	0.86	1.11
AT2G40490	uroporphyrinogen decarboxylase, putative	0.55	0.67
AT2G41560	calcium-transporting ATPase 4	0.44	0.68
AT2G47070	squamosa promoter-binding protein-like 1	0.52	1.10
AT2G47940	DegP2 protease	0.58	0.91
AT3G01380	phosphatidylinositolglycan class family protein	0.42	0.79
AT3G03930	protein kinase-related	0.78	0.90
AT3G12580	heat shock protein 70, putative	1.08	1.09
AT3G13360	WPP-domain interacting protein 3	0.56	1.48
AT3G18290	zinc finger protein-related	0.39	0.66
AT3G18570	glycine-rich protein/oleosin	3.23	N/D
AT3G22370	alternative oxidase 1a, mitochondrial (AOX1A)	1.40	1.38
AT3G23790	AMP-binding protein	0.40	0.75
AT3G47470	chlorophyll A-B binding protein 4, chloroplast	0.17	0.41
AT3G50370	expressed protein	0.52	0.86
AT3G53230	CDC48 – like protein	0.81	0.81
AT4G05420	UV-damaged DNA-binding protein, putative	0.53	0.84
AT4G09600	gibberellin-responsive protein 3	2.53	1.90
AT4G09980	methyltransferase MT-A70 family protein	0.51	0.78
AT4G27170	2S seed storage protein 4	1.31	1.25
AT4G27260	auxin-responsive GH3 family protein	0.89	1.11
AT4G27450	expressed protein	0.24	0.40
AT4G30530	defense-related protein, putative	0.78	0.81
AT4G30650	low temperature and salt responsive protein	3.62	1.33
AT4G31500	cytochrome P450 83B1 (CYP83B1)	0.60	0.77
AT4G36010	pathogenesis-related thaumatin family protein	1.61	1.79
AT4G38350	patched family protein	0.68	0.87
AT4G38600	ubiquitin-transferase family protein	0.67	0.95
AT4G39950	cytochrome P450 79B2, putative (CYP79B2)	0.49	0.70
AT5G04590	sulfite reductase/ferredoxin (SIR)	0.72	1.01
AT5G12030	cytosolic small heat shock protein	1.57	1.16
AT5G15450	heat shock protein 100, putative	0.93	1.51
AT5G16010	steroid 5alpha-reductase-like protein	1.12	1.16
AT5G17020	exportin1 (XPO1)	0.61	0.86
AT5G17790	VARIEGATED 3	0.43	0.85
AT5G19090	heavy-metal-associated domain protein	0.72	0.98
AT5G20700	senescence-associated protein-related	0.66	0.87
AT5G24570	expressed protein	1.11	0.96
AT5G28370	pentatricopeptide repeat-containing protein	0.73	1.07
AT5G44720	molybdenum cofactor sulfurase family protein	0.71	0.89
AT5G46070	guanylate-binding family protein	0.46	1.02
AT5G46580	pentatricopeptide repeat-containing protein	0.60	0.80
AT5G48850	male sterility MS5 family protein	1.39	1.76
AT5G51440	mitochondrial heat shock 22 kd protein-like	1.25	0.98
AT5G54740	protease inhibitor/seed storage family protein	0.82	0.94
AT5G58375	expressed protein	0.85	1.02
AT5G61780	nuclease family protein	0.62	0.76
AT5G63135	expressed protein	0.65	1.23
AT5G64230	expressed protein	1.26	1.24
AT5G64310	arabinogalactan-protein	5.52	3.36
AT5G64460	expressed protein	0.63	0.70
AT5G66310	kinesin motor family protein	0.75	N/D
ATCG00220	PSII low MW protein	1.05	1.06

### Quantitative real-time RT-PCR validation of microarray data

To re-test the results obtained through microarray experiments, qRT-PCR analysis was performed on selected genes, with an emphasis placed on genes related to auxin transport and metabolism. Two biologically independent batches of Col wild-type, *sis7-1 *and *sis7-3 *seeds were sown on minimal media, incubated for 20 h and then transferred to 100 mM Glc or 100 mM sorbitol media and incubated for an additional 13 h under continuous light before harvest. Total RNA was extracted for qRT-PCR analysis to monitor the relative expression levels of an array of genes, including *PIN1*, *PIN3*, *CYP83B1*, *CYP79B2 *and *AT4G27260 *(an IAA-amido synthase). On 100 mM sorbitol (osmotic control) media, the expression levels of these genes are similar in *sis7-1*, *sis7-3 *and Col wild type. In contrast, on 100 mM Glc media, the transcript levels of each gene are lower in the *sis7-1 *and *sis7-3 *mutants than in the Col wild type (Figure 8). These qRT-PCR results confirm the findings from microarray data that the steady-state mRNA levels of genes involved in auxin transport and metabolism are lower in the *sis7 *mutants than in the wild type on 100 mM Glc.

### *sis7-1 *mutants exhibit increased lateral root systems

**Figure 9:**
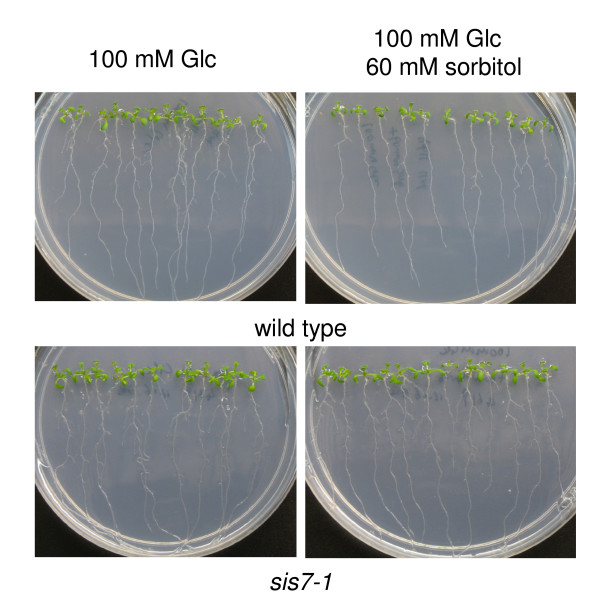
***sis7-1 *exhibits increased root system size on sorbitol media**. Wild-type Col and *sis7-1 *seedlings were grown for 12 d on vertically-oriented Petri plates containing the indicated media. Root systems of *sis7-1 *are less repressed by osmotica than are the root systems of wild-type seedlings.

The decreased mRNA levels of auxin-related genes observed in Glc-treated *sis7-1/nced3-4/sto1-4 *seeds provide further evidence of cross-talk between these pathways. To test whether the osmotic regulation of lateral root development is altered in the *sis7 *mutants, wild-type and *sis7-1/nced3-4/sto1-4 *seeds were germinated and grown on sorbitol media. The *sis7-1 *seedlings exhibit increased lateral root systems on sorbitol, when compared to the wild-type plants (Figure 9). These observations are consistent with previous findings that *aba2-1 *and *aba3-1 *display increased root system size on mannitol [48].

### Evidence for an expanded role for ABI3 in sugar response through mapped-based cloning of *SIS10*

The *sis10-1 *mutant was isolated during the same screen of a T-DNA mutagenized population, described above, that was used to isolate the *sis7-1 *and *sis7-3 *mutants. The *sis10-1 *mutant has strong Glc and Suc insensitive phenotypes during early seedling development. A map-based approach was used to identify the *SIS10 *gene. Towards this end, *sis10-1 *(in the Col-0 ecotype) was crossed with a wild-type plant of the Hi-O ecotype. F2 seeds from this cross were selected using *Arabidopsis *media supplemented with 320 mM Suc to identify seedlings with a *sis *mutant phenotype. An F2 population of 320 *sis *plants was then analyzed using SSLP markers. The *sis10-1 *mutation was mapped to chromosome 3, approximately 0.15 cM above marker 470509 on BAC MSD24. Examination of this region of the genome revealed that *ABI3 *(At3g24650) is present in the region believed to contain the *sis10 *locus. Sequencing of *ABI3 *DNA isolated from *sis10-1 *revealed that the *sis10-1 *mutation results in deletion of 16 nucleotides between base pairs 2715 and 2730 of the genomic DNA (with respect to the start codon) in the 6^th ^exon of *ABI3*. Accordingly, the *sis10-1 *mutant has been renamed *abi3-15*.

**Figure 10:**
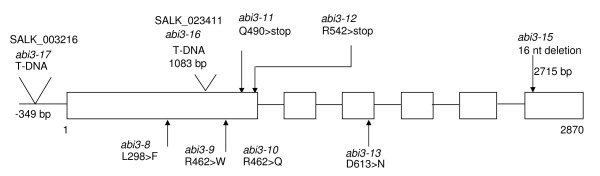
**Molecular lesions present in different *abi3 *mutants**. Nucleotides are numbered with respect to the start codon. Exons are depicted with boxes and introns are depicted with single lines.

**Figure 11:**
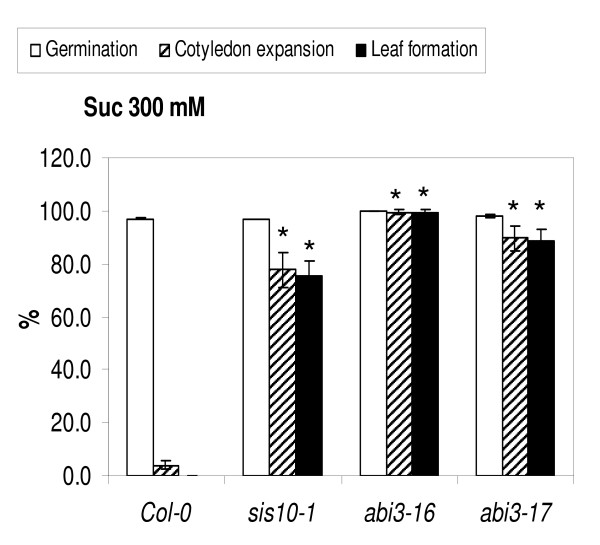
**Suc-response assays of three *abi3 *mutants**. Seeds were sown on media containing 300 mM Suc and incubated at 22°C for 14 d under 2200-3400 Lux continuous light prior to scoring. Seed germination, cotyledon expansion and true leaf formation were scored as previously described [16]. Error bars indicate standard deviations. Asterisks indicate results where the mutant phenotype differed from the corresponding wild-type phenotype with a *p*-value of less than 0.05 according to a Student's t-test.

Previous work from several labs [11,13,16] characterizing the *abi3-1 *mutant, which is in the Landsberg *erecta *background, found only slight to no effect of this mutation on sugar response. Therefore, the finding that the *sis10-1 *mutation, which causes a strong *sis *phenotype, lies in the *ABI3 *gene was unexpected. To test whether the mutation in *ABI3 *is truly the cause of the *sis10-1 *sugar insensitive phenotype, two additional *abi3 *mutant lines were obtained and characterized. SALK_023411 carries a mutation, here designated as *abi3-16*, consisting of a T-DNA insertion in the first exon of the *ABI3 *gene (Figure 10). SALK_003216 carries a mutation, here designated as *abi3-17*, consisting of a T-DNA insertion in the *ABI3 *promoter region [50]. Sugar-insensitivity assays on plants homozygous for the *sis10-1/abi3-15*, *abi3-16 *and *abi3-17 *mutations reveal that all three mutations confer significant resistance to the inhibitory effects of 300 mM Suc on early seedling development, compared to wild-type plants (Figure 11). These results confirm that mutations in *ABI3 *can lead to a strong sugar-insensitive phenotype.

**Figure 12:**
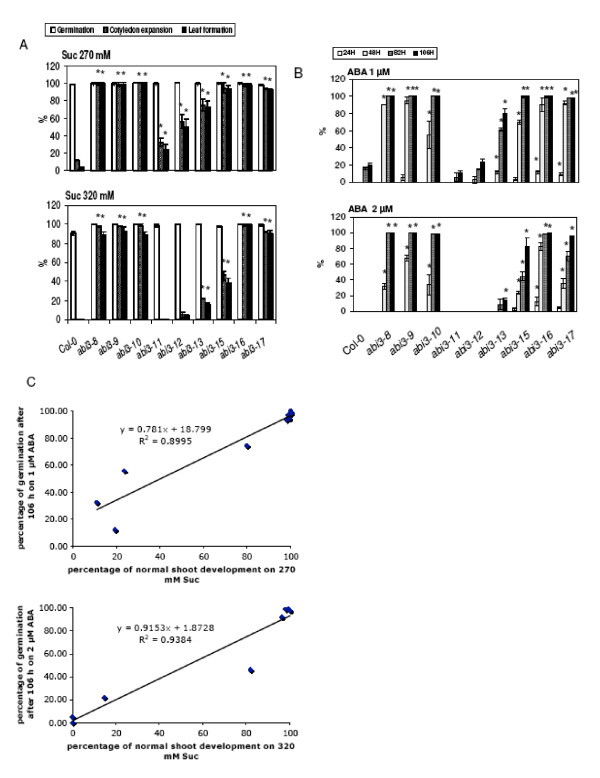
**Quantification of Suc and ABA insensitivity in nine *abi3 *mutants**. (A) For Suc-response assays seeds were sown on media containing 270 or 320 mM Suc and incubated at 22°C for 14 d under 2200-3400 Lux continuous light prior to scoring. Seed germination, cotyledon expansion and true leaf formation were scored as previously described [16]. Error bars indicate standard deviations. Asterisks indicate results where the mutant phenotype differed from the corresponding wild-type phenotype with a *p*-value of less than 0.05 according to a Student's t-test. (B) For ABA response assays seeds were imbibed on media with the indicated ABA concentrations and scored for germination after 24, 48, 82 and 106 h. Seed germination is defined as the emergence of the radicle from the seed coat. Error bars indicate standard deviations. Asterisks indicate results where the mutant phenotype differed from the corresponding wild-type phenotype with a *p*-value of less than 0.05 according to a Student's t-test. (C) Regression analysis of data on Suc and ABA response assays reveals strong positive correlation between sugar- and ABA- insensitivity. Each data point on the scatterplot represents the percentage of normal shoot development (defined as seedlings with green expanded cotyledons) on Suc media and the percentage of seeds that germinated after 106 h on ABA media for each genotype. Correlation coefficients were calculated for two assay conditions: 1 μM ABA and 270 mM Suc representing relatively non-stringent assay conditions and 2 μM ABA and 320 mM Suc representing more stringent assay conditions.

Previous work indicated that *abi3-1 *has a relatively strong ABA insensitive seed germination phenotype [28], but displays only a very subtle sugar insensitive phenotype during early seedling development [11,13,16]. The finding that other *abi3 *mutants, such as *sis10-1/abi3-15*, *abi3-16 *and *abi3-17*, can have strong sugar-insensitive phenotypes during early seedling development raised the possibility that different mutations in *ABI3 *may predominantly affect either ABA or sugar response. To test this hypothesis a careful analysis was conducted to determine the relative degrees of sensitivity to the inhibitory effects of ABA on seed germination and of Suc on early seedling development of nine different *abi3 *mutant lines (Figure 12). These nine *abi3 *lines included the *sis10-1/abi3-15*, *abi3-16 *and *abi3-17 *mutants described above, as well as the *abi3-8, abi3-9, abi3-10, abi3-11, abi3-12 *and *abi3-13 *mutants [23]. All of these mutants are in the Col ecotype. The molecular lesions of the different *abi3 *mutants are depicted in Figure 10. To measure sugar response, seeds of all nine *abi3 *mutants plus Col wild-type were sown on minimal *Arabidopsis *media [31] supplemented with 270 or 320 mM Suc and grown in continuous light for 14 d before scoring. These Suc concentrations were chosen to allow detection of differences in the degree of Suc insensitivity. The 270 mM Suc concentration is sufficient to allow detection of mutants with relatively weak sugar-insensitive phenotypes, as these mutants will still exhibit a significantly greater ability than wild-type plants to develop expanded cotyledons and true leaves on 270 mM Suc. However, a 270 mM concentration of Suc may not be sufficient to distinguish between mutations that confer moderate versus strong sugar-insensitive phenotypes. This distinction can be made using the 320 mM Suc concentration. Selection on media supplemented with 320 mM Suc revealed that five of the *abi3 *mutations (*abi3-8, abi3-9, abi3-10, abi3-16 *and *abi3-17*) confer strong sugar-insensitive phenotypes. The other *abi3 *mutations tested conferred lesser degrees of sugar-insensitivity. The relative degree of sugar insensitivity of these other four mutants, from highest to lowest, is: *abi3-15, abi3-13, abi3-12 *and *abi3-11 *(Figure 12A). To assess the relative effects of the different *abi3 *mutations on ABA sensitivity, seed germination was assayed at different time points and in the presence of different concentrations of ABA (Figure 12B). A regression analysis of the results indicates a strong positive correlation between sugar insensitivity and ABA insensitivity of tested *abi *mutants (Fig 12C). In other words, mutations that confer a high degree of insensitivity to ABA also confer a high degree of insensitivity to Suc. Conversely, mutations that confer only weak ABA insensitivity also confer weak insensitivity to Suc.

## Discussion

Molecular genetic studies on *Arabidopsis *sugar response mutants have revealed extensive evidence for cross-talk between sugar and phytohormone response pathways [11-13,15-18,51,52]. For example, exogenous Glc has been proposed to slow the decrease in ABA concentrations that occurs during seed germination [53]. Glc has also been shown to help regulate expression of a number of genes involved in ABA metabolism in seedlings. Several ABA biosynthetic genes, including *ABA1*, *AAO3 *and *ABA3 *are upregulated by 110 mM and 330 mM Glc via a mechanism that requires that a certain endogenous ABA level is maintained [17]. Interestingly, these same genes are downregulated by 330 mM mannitol, via a mechanism that does not appear to require wild-type levels of endogenous ABA. These results suggest that regulation of these three genes by Glc is distinct from their regulation by osmotic stress. Similarly to *ABA1, AAO3 *and *ABA3*, the ABA biosynthetic gene *ABA2 *is also upregulated by 110 mM and 330 mM Glc via a mechanism that requires that endogenous ABA levels be maintained above a certain level. However, unlike *ABA1, AAO3 *and *ABA3*, expression of *ABA2 *is also upregulated by 110 mM and 330 mM mannitol via a mechanism that does require maintenance of endogenous ABA levels [17]. So, although regulation of all four genes by Glc appears similar, *ABA2 *exhibits significant differences in expression in response to osmotic stress.

The ABA biosynthetic gene *SIS7/NCED3/STO1 *also exhibits an expression pattern that is distinct from that of the other ABA biosynthetic genes discussed above. In wild-type seedlings, the expression of *SIS7/NCED3/STO1 *is not activated by Glc but is strongly induced by salt or osmotic stress [17,26]. In fact, SIS7/NCED3/STO1 may act primarily in the biosynthesis of ABA during stress conditions [29]. Due to these regulatory and functional differences, it was not clear whether mutations in *SIS7/NCED3/STO1 *would cause a sugar-insensitive phenotype, as had been previously shown for mutations in other ABA biosynthetic genes [11,13,16]. Results presented here, showing that the *sis7 *mutations lie in *NCED3/STO1*, demonstrate that mutations in *SIS7/NCED3/STO1 *do confer a strong sugar-insensitive phenotype.

Expression of *SIS7/NCED3/STO1 *has been suggested to be rate-limiting in the positive feedback regulation of ABA biosynthesis by ABA under stress conditions [54,55]. Transcriptional upregulation of *SIS7/NCED3/STO1 *by ABA has been found in certain ABA-deficient mutants and ecotypes [17,26]. However, the transcriptional regulation of *NCED3 *as well as other ABA biosynthesis genes by Glc in germinating seeds is unclear. Results presented here (Figure 6) show that in wild-type germinating seeds high concentrations of exogenous Glc result in increased *SIS7/NCED3 *steady-state mRNA levels. Steady-state mRNA levels of *ABA1*, *ABA2 *and *ABA3 *in wild-type germinating seeds are also upregulated by high concentrations of exogenous Glc. In contrast, in germinating seeds of the *sis7-1 *and *sis7-3 *mutants, Glc induction of *ABA1*, *SIS7/NCED3*, *ABA2 *and *ABA3 *is abolished. Previously it has been shown that 333 mM Glc induces ABA accumulation (or decreases ABA metabolism) in imbibed seeds [53]. The results of experiments presented here, analyzing Glc regulation of ABA biosynthesis genes in *sis7 *and wild-type germinating seeds, indicate that high Glc levels activate ABA biosynthesis by increasing the steady-state mRNA levels of ABA biosynthesis genes. These increases may, in turn, subsequently lead to ABA accumulation.

Besides transcriptional regulation, translational and post-translational mechanisms may also be involved in sugar-mediated regulation of ABA levels and/or signaling in germinating seeds. For example, loss-of-function of the *KEEP ON GOING *(*KEG*) gene, which is involved in ABI5 degradation, causes the mutant seedlings to exhibit a sugar hypersensitive phenotype [56]. In wild-type germinating seeds, the Glc-mediated increases in ABA levels may induce *SIS7/NCED3 *expression, as well as the expression of other ABA biosynthetic genes, thus resulting in sustained increases in ABA levels [26,55]. In the *sis7/nced3/sto1 *mutant, this positive feedback control loop is likely broken as is evidenced by the loss of Glc induction of *ABA1*, *SIS7/NCED3*, *ABA2 *and *ABA3 *in *sis7-1 *and *sis7-3 *germinating seeds. Thus the failure to accumulate ABA under 'sugar stress' may render mutant seedlings unable to carry out a normal sugar response. Consistent with this possibility are findings that the *nced3/sto1 *mutant accumulates only one-third as much ABA as wild type under sorbitol stress [27]. In addition, it has been reported that ABA levels in *sto1/nced3 *plants are somewhat lower than those found in unstressed wild-type plants [27]. These results may explain the enhanced germination rate of *sis7-1 *seeds on very low (1 μM), but not on higher (2-5 μM), levels of ABA. Also, addition of exogenous ABA to the 220 mM Glc media eliminated the sugar-insensitive phenotype of *sis7/nced3/sto1 *seedlings, indicating that the *sis *phenotype is caused by a deficiency in endogenous ABA.

As some mutants that are resistant to Glc and Suc also exhibit resistance to Man, the ability of the *sis7-1 *mutant to germinate on media containing 1.5 mM and higher concentrations of Man was tested. Man inhibition of seed germination has been postulated to act through a hexokinase-dependent pathway. This hypothesis is based on the fact that the hexokinase inhibitor mannoheptulose can reverse the inhibitory effects of Man on germination of wild-type seeds [35]. The results of experiments testing the ability of *sis7-1 *seeds to germinate in the presence of Man indicate that *sis7-1 *does not exhibit increased resistance to the inhibitory effects of exogenous Man on seed germination. This finding is consistent with the results of Man seed germination assays conducted on other *aba *mutants, where *aba1-1 *and *aba3-2 *were found to be unable to germinate in the presence of Man and *aba2-1 *[13] and *aba2-3 *and *aba2-4 *[16] were found to be only very slightly resistant to Man. These results suggest that response to the inhibitory effects of Man on seed germination may not require ABA accumulation. In contrast, mutations in *ABI4 *and *ABI3 *cause reduced seed sensitivity to Man [24,35], indicating that defects in ABA signaling components can affect Man sensitivity. The molecular mechanism by which defects in some ABA signaling components, but not defects in components of the ABA biosynthetic pathway, affects inhibition of seed germination by Man remains to be elucidated. Further complicating analyses of the role of *SIS7/NCED3/STO1 *in seed germination and other processes are findings that *SIS7/NCED3/STO1 *belongs to a multigene family with nine members in *Arabidopsis*, five of which are postulated to be involved in ABA biosynthesis [37,57].

Transcriptional profiling analyses identified 83 genes with altered mRNA levels in *sis7-1 *germinating seeds grown on 100 mM Glc. Functional categorization and over-representation analyses by GO annotations revealed significant enrichment of genes involved in response to stimulus, particularly abiotic stimulus. An interesting finding from these analyses is the decreased steady-state mRNA levels of auxin biosynthesis and transport genes in *sis7-1 *treated with Glc when compared to the wild type. These findings were re-tested by qRT-PCR analyses. The results of the qRT-PCR experiments confirm that the steady-state mRNA levels of these auxin-related genes are indeed lower in *sis7-1 *and *sis7-3 *germinating seeds compared to wild-type seeds (Figure 8). Previous studies have suggested cross-talk between the ABA and auxin response pathways in regulating *Arabidopsis *root system development [47-49]. The *sis7-1 *mutant exhibits increased lateral root development on sorbitol media when compared to the wild type (Figure 9). In addition, *AtNCED3::GUS *expression has been observed at lateral root initiation sites, suggesting control of lateral root development by both ABA and auxin [37]. The findings reported here suggest that part of the molecular mechanism(s) by which ABA/auxin cross-talk occurs during regulation of root development may involve regulation of auxin biosynthesis and transport genes in response to ABA levels.

To determine which of the genes listed in Table 1 are glucose regulated, the results from GeneChip experiments performed using seeds grown in the presence of 100 mM Glc were compared with data from GeneChip experiments performed using seeds grown in the presence of equimolar sorbitol (Table 2). Interestingly, a subset of the 83 genes listed in Table 1 exhibit only slight or no obvious Glc response in wild-type seeds, but exhibit significant Glc regulation in *sis7-1 *seeds, which have decreased ABA levels [27,29]. This result suggests that changes in endogenous ABA levels modulate the expression of this subset of genes. Modulation of Glc response by ABA at transcriptional or tissue levels has been demonstrated. Rook et al. (2001) have shown that ABA does not activate *ApL3 *expression alone, but greatly enhances *ApL3 *induction by sugars [18]. Glucose has been shown to delay *Arabidopsis *seed germination [53,58]. Dekkers et al. (2004) have demonstrated that low ABA concentrations combined with Glc enhance the inhibitory effect of Glc on seed germination [58]. Together, these results indicate that Glc-response may be modulated by ABA levels.

Different *abscisic acid insensitive *(*abi*) mutants display different responses to high levels of exogenous Glc and Suc. Previous studies showed that mutations in *ABI4 *confer significant resistance to the inhibitory effects of high concentrations of exogenous Glc or Suc on early seedling development, whereas the *abi1-1*, *abi2-1*, *abi3-1 *and *abi5-1 *mutants exhibit only slight to no resistance to Glc or Suc [11,13,16,18]. Interestingly, *abi3-6 *mutants, which lack one third of the *ABI3 *gene and are thus believed to be null mutants [59], have been shown to be resistant to the inhibitory effects of 330 mM Glc on early seedling development [25]. However, quantitative data indicating the degree to which *abi3-6 *mutants are resistant to Glc are lacking. Therefore, the finding that the *sis10-1 *mutant, which has a strong *sis *phenotype, lies in the *ABI3 *gene was unexpected. In addition, the findings reported here that *sis10-1/abi3-15 *has significant *sis *and *abi *phenotypes, whereas *abi3-1 *has been reported to have a significant *abi *phenotype [28] but little to no *sis *phenotype [11,13,16], suggested that it might be possible to separate genetically the role of ABI3 in ABA and sugar response. To test this hypothesis, quantitative assays were conducted to determine the sensitivities of nine different *abi3 *mutant lines, all in the Col background, at two Suc and two ABA concentrations. The results of these experiments indicate that the degree of Suc resistance shows a close, positive correlation with the degree of ABA resistance. These results therefore do not provide evidence for a genetically separable role for ABI3 in sugar and ABA resistance, although such a possibility cannot be ruled out at this time. The previous findings that *abi3-1 *has only a very slight effect on sugar response may be at least partially explained by the fact that the *abi3-1 *mutation is in the Landsberg *erecta *rather than the Col background. The Landsberg *erecta *ecotype exhibits a significantly greater level of sugar sensitivity than that of the Col ecotype (data not shown and [16]). A role for ABI3 in both sugar and ABA response is also consistent with results indicating that mutants carrying specific *abi3 *alleles exhibit increased resistance to Glc in the presence of ABA [23] and that overexpression of *ABI3 *confers hypersensitivity to Glc [60].

## Conclusion

In this paper the map-based cloning and characterization of two genes that affect sugar response at the early seedling developmental stage are reported. These studies resulted in identification of new *sis *mutants which carry defects in the ABA biosynthesis gene *NCED3/STO1 *or the ABA response gene *ABI3*. The *SIS7/NCED3/STO1 *gene is rate-limiting in the positive feedback regulation of ABA biosynthesis by ABA under stress conditions [17,26]. Transcriptional upregulation of ABA biosynthesis genes by 300 mM Glc has been shown to occur in germinating wild-type seeds, but not in germinating *sis7-1 *or *sis7-3 *seeds. Transcriptional profiling experiments resulted in identification of 83 transcripts with altered steady-state mRNA levels in *sis7-1 *versus wild-type germinating seeds treated with 100 mM Glc. Over-representation analysis revealed that these 83 genes are significantly enriched for genes involved in response to abiotic stimulus. Of particular interest are findings that auxin metabolism and transport genes are significantly over-represented in genes with lower transcript levels in the *sis7-1/nced3-4/sto1-4 *mutant. These results have been verified by qRT-PCR analyses. These findings suggest that part of the molecular mechanism(s) by which ABA and auxin interact during regulation of *Arabidopsis *root development may involve regulation of auxin homeostasis and/or transport by ABA levels. Previous reports that *abi3-1 *has a significant *abi *phenotype but little to no *sis *phenotype, together with results presented here showing that *sis10-1/abi3-15 *has significant *sis *and *abi *phenotypes, suggested that it might be possible to separate genetically the role of ABI3 in sugar and ABA response. However, quantitative analyses of the magnitudes of the defects in Suc and ABA response of nine different *abi3 *mutants, all in the Col background, show that the strengths of these two phenotypes exhibit a strong positive correlation. The relatively weak *sis *phenotype shown by the *abi3-1 *mutant may be due, at least in part, to the fact that it is in the Landsberg *erecta *background, as different ecotypes have been shown to exhibit significantly different levels of endogenous sugar sensitivity (data not shown and [16]).

## Methods

### Plant materials, growth conditions and media

The *sis7-1*, *sis7-3 *and *sis10-1 *mutants were isolated from a mutant population generated by insertion of a T-DNA library carrying random *Arabidopsis *cDNAs into the genome of *Arabidopsis thaliana *of the Columbia (Col) ecotype [34]. The *sis7-2 *mutant was obtained by screening ethyl methyl sulfonate (EMS)-mutagenized *Arabidopsis thaliana *var. Col from Lehle Seeds (Tucson, AZ, USA). The *gl1 *mutation was removed from the *sis7-2 *mutant line by backcrossing this line to wild-type plants of the Col ecotype. The *abi3-16 *(SALK_023411) and *abi3-17 *(SALK_003216) mutants were identified from a T-DNA mutagenized population of *Arabidopsis thaliana *var. Col [50]. Seeds that are homozygous for the *abi3-16 *mutation were obtained from Drs. Laurie Host and Mauricio Bustos (University of Maryland, Baltimore County). Seeds segregating for the *abi3-17 *mutation were obtained from the Arabidopsis Biological Resource Center. PCR was then used to screen for plants that are homozygous for the *abi3-17 *mutation. The *abi3-8*, *abi3-9*, *abi3-10*, *abi3-11*, *abi3-12 *and *abi3-13 *mutants [23] of *Arabidopsis thaliana *var. Col were obtained from Dr. Eiji Nambara (RIKEN Plant Science Center, Japan). Isolation of *abi4-103*, which is in the Col background, has previously been described [16]. Seeds of *spy-3 *[36], which is also in the Col background, were obtained from Dr. Neil Olszewski. Wild-type Hi-O (CS6736) and Col-0 (CS6000) seeds were obtained from the Arabidopsis Biological Resource Center at Ohio State University. Seeds to be sown on plates were surface-sterilized and stratified in the dark at 4°C for 3 d. Plants were grown on plates under continuous white fluorescent light at 22°C unless otherwise noted. Minimal *Arabidopsis *media was prepared as described [31].

### Sugar-insensitive mutant screen and genetic analyses

To isolate sugar-insensitive mutants, M2 seeds derived from EMS-mutagenized Col seeds were sown on Petri plates containing minimal media supplemented with 300 mM Suc. Seeds derived from a T-DNA insertion library [34] were screened on minimal media supplemented with 340 mM Suc. Subsequent screening procedures were performed as described [16]. All *sis *mutations were caused by single locus recessive mutations (data not shown). The *sis7-1/nced3-4/sto1-4 *mutant was backcrossed twice and the *sis7-2/nced3-5/sto1-5 *mutant was backcrossed once to wild-type Col before physiological assays were conducted.

### Sugar response assays

The sugar sensitivity of the mutants was assayed as described previously [16].

### Abscisic acid and paclobutrazol response assays

To measure ABA sensitivity, seeds were sown on minimal media supplemented with 0, 1, 2 or 5 μM ABA. Plates were incubated at 25°C under continuous light. The percentages of seeds that had germinated were determined at time points of up to 9 d. Germination is defined as the emergence of the radicle from the seed coat. To measure paclobutrazol sensitivity, seeds were sown on minimal media supplemented with 0, 30, 60 or 240 μM paclobutrazol. Plates were incubated at 25°C under continuous light. The percentages of seeds that had germinated were determined until the eighth d.

### Map-based cloning

Mapping populations for *sis7-1 *and *sis10-1 *were generated as described in the Results section. Each of these F2 mapping populations was screened on media containing 300-340 mM Suc to identify plants that are homozygous for the *sis *mutation carried within that population. Genomic DNA was prepared from leaves of these seedlings via alkaline lysis [61]. Other mapping procedures were performed as described [62,63]. The molecular markers used to identify recombinants in the mapping experiments were simple sequence length polymorphisms (SSLPs), cleaved amplified polymorphic sequences (CAPS) and single nucleotide polymorphisms (SNPs) that were obtained from the The Arabidopsis Information Resource (TAIR) and Monsanto SNP and Ler sequence collections [62] and tested for polymorphisms between Col and Hi-O wild-type plants.

### Reverse Transcriptase (RT) PCR Assays

The Qiagen RNeasy plant mini kit (Qiagen, Valencia, CA) was used to isolate total RNA from leaves of 4-week-old wild-type Col and *sis7 *mutant plants grow on soil under continuous light. First strand cDNA was synthesized from total RNA (0.5 μg) using the Promega Improm-II RT system kit (Promega, Madison, WI). Two μl of cDNA were used for PCR reactions. Primers used were: *SIS7*, 5'-TAAAAACCGTTGGTCGGTTC-3' and 5'-CGACGTCCGGTGATTTAGTT-3'; *ACTIN*, 5'-TGCTGACCGTATGAGCAAAG-3' and 5'-GATTGATCCTCCGATCCAGA-3'.

### Quantitative Real-Time PCR

For Glc regulation of ABA biosynthesis gene expression, seeds of Col, *sis7-1 *and *sis7-3 *were stratified and sown on nytex screens on minimal media and incubated for 20 h under continuous light and then transferred to 300 mM Glc or sorbitol media for an additional 12 h under continuous light before harvesting. For validation of microarray data, seeds of Col, *sis7-1 *and *sis7-3 *were treated as described above, except that the seeds were transferred to 100 mM Glc or sorbitol media. Seeds from two biologically independent batches were used to minimize the variations in gene expression engendered by possible changes in plant growth conditions. Total RNA was isolated using the Spectrum™ Plant Total RNA Kit (Sigma). First strand cDNA was synthesized with the Promega Improm-II RT system kit (Promega) in a reaction volume of 20 μl. Real-time PCR was performed on an Applied Biosystems 7500 Real-Time PCR Machine using the SYBR Green JumpStart Taq ReadyMix (Sigma, St. Louis, MO). *UBQ6 *(AT2G47110) was used as an internal reference. The following primers were used: *UBQ6*, 5'-CCATCGACAATGTCAAGGCC-3' and 5'-GGTACGTCCGTCTTCGAGCT-3'; *ABA1*, 5'-GAACGTACTATAAAGGGAGAATGG-3' and 5'- CTGAGACGAAGGGATCACAAT-3'; *SIS7/NCED3*, 5'-TAAAAACCGTTGGTCGGTTC-3' and 5'-CGACGTCCGGTGATTTAGTT-3'; *ABA2*, 5'-CTAAACTCGCTTTGGCTCATT-3' and 5'-CGCTACATCATCAACCGTCAG-3'; *ABA3*, 5'-TTTGCTGGGATGACAATGAT-3' and 5'-CCTTCCACTGACGACGGTTC-3'; *AAO3*, 5'-GTCAGCGAGGTGGAAGTGGA-3' and 5'-CAAATGCTCCTTCGGTCTGT-3'; *CYP83B1*, 5'-CCCTAACCGCCCTAAACAAGA-3' and 5'-CATACCACCACTGCAGCCG-3'; *CYP79B2*, 5'-GACAATCCATCAAACGCCGT-3' and 5'-GCGGAGGATAGCTTTGACGTA-3'; *AT4G27260*, 5'-TGTTGTCACCACTTACGCCG-3' and 5'-CGTTCTGAAGCTCAACCTCGT-3'; *PIN1*, 5'-CGGTGGGAACAACATAAGCA-3' and 5'-TGAAGGAAATGAGGGACCAGG-3' and *PIN3*, 5'-TGGAGATTTCGGAGGAGAACA-3' and 5'-TTCGTTGACTTGCTTCGGC-3'.

### Gene expression analysis using Affymetrix ATH1 GeneChips

For transcriptional profiling experiments, Col wild-type and *sis7-1 *seeds were sown on nytex screens on minimal media and incubated for 20 h under continuous light and then transferred to 100 mM Glc or sorbitol media for an additional 13 h under continuous light before harvesting. Seeds were pooled from different batches to minimize the variation in gene expression profiles engendered by possible changes in plant growth conditions. Total RNA extraction was performed as described [64]. For reproducibility, three independent seed tissue collections and RNA extractions were performed for *sis7-1 *treated with Glc or sorbitol and Col wild type treated with sorbitol and six independent experiments were performed for Col wild type treated with Glc. The generation of biotinylated cRNA from total RNA and subsequent hybridization to *Arabidopsis *ATH1 genome arrays and quantification of fluorescent signals were performed by the Molecular Genomics Core Facility at the University of Texas Medical Branch at Galveston. Data analyses were performed using Expressionist (Genedata AG, Switzerland). Gene expression values and presence/absence calls were computed using the Affymetrix Statistical Analysis (MAS5) algorithm. Student's t-test values and fold changes of individual transcript signal intensities from *sis7-1 *and wild-type replicates were determined. The mis-regulated gene list was generated by identifying transcripts that varied between mutant and wild-type samples with a *p *value of less than 0.05 and a fold change value of greater than 2.0. For over-representation analysis using GeneMerge, the population genes consist of 8791 genes called present on the ATH1 arrays by the Expressionist program. *P *value was calculated after Bonferroni correction.

## Authors' contributions

YH identified the *SIS7/NCED3/STO1 *gene, performed most of the characterization of the *sis7/nced3/sto1 *mutants and drafted the major part of the manuscript. CL identified the *SIS9/ABA1 *and *SIS10/ABI3 *genes, performed the characterization of the *sis10/abi3 *mutants and aided in drafting the manuscript. KB identified the *sis7-1, sis7-3 *and *sis10-1 *mutants and performed a preliminary characterization of those mutants. SG conceived of the study, assisted in its design and the interpretation of the data and aided in drafting the manuscript. All authors approved of the manuscript.

## Note

During submission of this manuscript, a manuscript appeared (Plant Mol Biol 67: 151) that also describes findings that mutations in ABI3 can confer a sugar-insensitive phenotype.
